# Rapidly Progressive Osteoarthritis of the Hip: A Prospective Study

**DOI:** 10.3390/jcm13092467

**Published:** 2024-04-23

**Authors:** Luis Angel Montero Furelos, Alberto De Castro Carrasco, Santiago Cons Lamas, Francisco Borja Sanchez Sierra, José R. Caeiro-Rey

**Affiliations:** Orthopedic Surgery and Traumatology Service, Hip Unit, Santiago de Compostela University Clinical Hospital, SERGAS, 15706 Santiago de Compostela, Spain; alberto.de.castro.carrasco@sergas.es (A.D.C.C.); santiago.cons.lamas@sergas.es (S.C.L.); francisco.borja.sanchez.sierra@sergas.es (F.B.S.S.); jose.ramon.caeiro.rey@sergas.es (J.R.C.-R.)

**Keywords:** rapidly progressive osteoarthritis of the hip (RPOH), risk factor, total hip arthroplasty, osteoarthritis (OA), systematic review

## Abstract

**Background:** Rapidly progressive osteoarthritis of the hip (RPOH) is a rare syndrome that involves the femoral head and acetabulum. **Methods:** We analyzed the incidence of RPOH in 2022. The inclusion criteria included a clinical history of pain for 1–6 months and a decrease in joint space of > 2 mm within one year or a decrease in joint space by 50% in that time accompanied by femoral and/or acetabular bone destruction. Exclusion: There are no other destructive arthropathies and no evolutionary radiological image sequence. **Results:** There were 15 patients, 16 hips, an incidence around 3.17%, a 1:1 laterality ratio, and 1 bilateral affected. The mean average age is 77.35 years. The male/female ratio is 1:2. The average BMI is 31.2. The time of the onset of the symptoms to the patient’s diagnosis is 5 months. The functionally modified Harris scale (MHS) had an average score of 30 points. They had surgery hip arthroplasty with a cementless cup in all cases, a revision cup in one of them, and a double mobility cup in the other, with the stem cemented three times. There were no post-surgical complications. Functionally was achieved at 3 months. The average MHS is 70 at 12 months. The average MHS is 85. **Conclusions:** RPOH is an idiopathic entity characterized by great clinical involvement and rapid radiological evolution. It is most common in women around 77 years of age. The bone quality requires surgical alternatives to implants, and it has good functional recovery post-surgery.

## 1. Introduction

Rapidly progressive osteoarthritis of the hip (RPOH) is a rare syndrome involving the femoral head and acetabulum, first described by Forestier in 1957 [1]. Although its description is attributed to Lequesne [2], it was Postel and Kerboul [3] in 1970 who coined the name rapidly destructive osteoarthritis. It is characterized by the destruction of the hip joint in a short time. It has characteristics common to primary hip osteoarthritis and to avascular necrosis.

Currently, there is no standardized definition of the characteristics of this disease, with the definition proposed by Lequesne [2] being accepted in many cases as the loss of joint space of 2 mm per year or the loss of 50% of the joint space in one year, in the absence of another cause of destructive arthropathy. In the international literature, we can find it under various names: rapidly destructive hip disease, rapidly destructive arthropathy, rapidly destructive coxarthrosis, rapidly progressive idiopathic arthritis of the hip, or Postel’s disease [4].

Although its etiopathogenesis is unknown, immunological, vascular, or even systemic factors have been suggested since in some patients the involvement of other joints has been described [5].

This often proceeds to rapid bony destruction of the femoral head with or without acetabular involvement. It rarely occurs in patients with pre-existing osteoarthritis. A diagnosis of exclusion must be made, ruling out septic, inflammatory pathology; avascular necrosis; or neuropathic arthropathy.

There are few published series, and most of them are retrospective. Therapeutic management is surgical, performing total hip prostheses using cemented, hybrid, or uncemented implants. The short and medium-term clinical results are similar to those of primary coxarthrosis surgery [6] even though they are more complex surgeries with prolonged surgical time, special implants, and greater blood loss [7]. As there is a lack of incidence and general data for RPOH, we evaluate the calendar year RPOH patients in the cohort of hip osteoarthritis treated by total hip arthroplasty.

## 2. Materials and Methods

We followed up with patients undergoing surgery in a tertiary hospital center in a unit dedicated to hip pathology, analyzing the prevalence and the characteristics of patients who suffer rapidly progressive osteoarthritis of the hip (RPOH), assessed in our unit in 2022. The inclusion criteria as a diagnosis of RPOH included a clinical history of pain of 1–6 months, a decrease in joint space > 2 mm within 1 year, or a decrease in joint space of 50% in that time accompanied by femoral and/or acetabular bone destruction.

**Figure 1:**
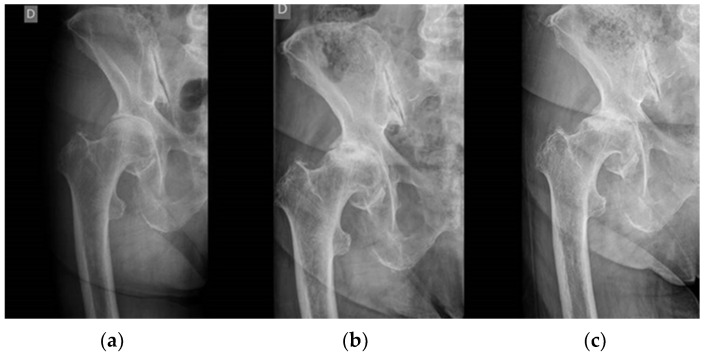
Radiological evolution of one of our patients 6 months after the onset of the first symptoms. (**a**), X-ray of the hip at the beginning of the symptoms; (**b**), radiographic study at 3 months and (**c**) at 6 months after the onset of symptoms. We can appreciate the rapid destructive evolution of the femoral head.

We analyze prospective primary osteoarthritis treated surgically in 2022, paying attention to those that met the inclusion criteria for a diagnosis of RPOH (Figure 1).

Exclusion criteria for patient selection were the existence of other destructive arthropathies (infectious, metabolic tumors, inflammatory or post-traumatic) and the non-existence of evolutionary radiological or clinical imaging sequence studies that mark the rapidly progressive evolution.

We analyzed the demographic characteristics, risk factors, clinical presentation, anatomopathological study, and surgical technique of patients diagnosed with RPOH.

## 3. Results

In patients undergoing total hip prosthetic surgery in 2022, we have diagnosed 15 patients (16 hips) with rapidly progressive hip osteoarthritis (RPOH). Within the 507 hip arthroplasties conducted in 2022 for hip osteoarthritis, there was an incidence of 3.17% for RPOH in our institution. With a 1:1 laterality relationship, one patient was affected in both hips.

The male/female ratio was 1:2 with an average age of 77.35 years and an average BMI of 31.2. Only 3 patients had a BMI greater than 35 (Table 1).

The time from the onset of symptoms to the diagnosis of patients has been an average of 5 months. Functionally, there was a modified Harris scale (MHS) score of less than 40 in all cases, with an average of 30 points. Six came to the consultation in a wheelchair (one patient was confined to the home), 5 came with crutches, and 4 came without aids.

The anesthetic risk according to the American Society of Anesthesiologists Physical Status (ASA) scale showed 7 patients rated as ASA III and 8 patients rated as ASA II. Taking into account the existence of cardiovascular risk factors, there were 10 hypertensive patients, 6 hypercholesterolemic patients on treatment with statins, and 5 patients with diabetes mellitus.

**Figure 2:**
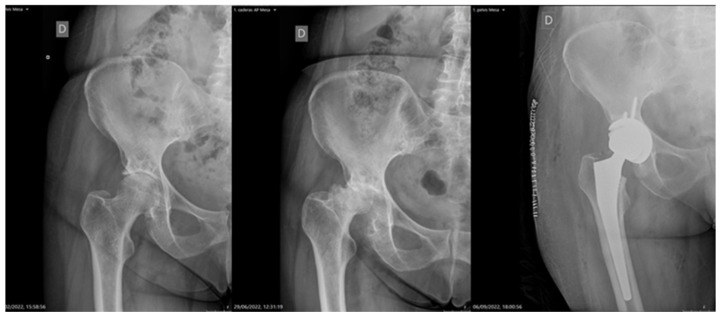
Patients with a torpid evolution who, after being included on the waiting list, went to the emergency department because of functional deterioration with poor pain control with opioid analgesics. Note the deterioration in the posterolateral angle, which is the most common pattern in our series.

They all underwent hip arthroplasty. A posterior approach was used with 14 hips using and an anterolateral approach was used with 2 hips The average surgical time was approximately 78 min. In all patients, the implantation of a cementless cup was performed at the acetabular level, with a revision cup in one of them and a double-mobility cup in another. At the femoral level, 13 patients had an implanted cementless stem (Figure 2) and a cemented stem in another 3. We used preoperative intravenous tranexamic acid in all of them in a dose of 1 g administered at the time of anesthetic induction, repeating doses depending on the bleeding, without requiring transfusion in any of the patients.

All femoral heads underwent anatomopathological study and presented characteristic areas with viable healthy tissue (viable zone), next to focal loci of osteonecrosis in subchondral bone (necrotic zone), and areas in bone repair (reparative zone), with the absence of a demarcation between healthy and necrotic tissue.

In the post-surgical evaluation, only one patient presented persistent wound drainage with spontaneous resolution. There were no post-surgical complications.

The short-term follow-up at 3 months functionally showed a mean MHS of 70 points. All patients were ambulant; 8 patients did not use aids for ambulation, 4 required a cane, and 4 patients required 2 canes. In the last review, the average time in the series was 13 months, including 20 months for the patient with the longest follow-up time and 9.5 months for the patient with the shortest follow-up. The mean functional assessment at the one-year follow-up according to the Harris scale was 85 points: all patients were ambulant—10 patients did not use aids for ambulation, 4 required a cane, and 1 patient required 2 canes.

## 4. Discussion

RPOH is a rare entity and is not well known, with a reported incidence of 7.2–15.7% of osteoarthritis. In our institution, there was an incidence of 3.17% for RPOH in 2022. Frequently, it is a woman with an average age of around 70 years, with a unilateral involvement in most cases (80–90%) characterized by great joint destruction in a period of 6–12 months from the onset of symptoms.

Some authors emphasize that rapid deterioration is more common in elderly and overweight women [8]. In our series, the involvement did not show any differences between sexes, with an age somewhat higher than that in the published series, around 77 years.

In our series, we report the clinical case of a male patient with bilateral RPOH.

On the other hand, we highlight a body mass index (BMI) in our series that is lower than that published—a BMI of 31.2, with only 5 patients above 35.

The etiology remains uncertain, suggesting several factors that could contribute to the development of this disease.

Genetic factors, primary osteoarthritis presents with hereditary components, being considered a polygenic disease. Genetic mutations can be directly related to the development of RPOH, which could determine the beginning of the disease, the affected joints, the severity of the involvement, and its speed of progression [9].

Mechanical factors have been related to RPOH: subchondral bone ischemia and subchondral insufficiency fracture are considered contributing factors to the development of this entity and have been observed on magnetic resonance images in the early stages [10]. Yamamoto and Bullough were the first to point out that subchondral insufficiency fracture can cause rapid rupture of the hip joint; however, it is not clear why the insufficiency fracture occurs [11].

Intraoperative findings have been described in which the anterosuperior portion of the acetabular labrum had inverted towards the joint space, with fractures observed due to subchondral insufficiency of the femoral heads just below the inverted labrum. This fact may be involved in the rapid narrowing of the joint space and in subchondral insufficiency fracture in RPOH [12].

Morphological factors associated with the appearance of RPOH have recently been described by some authors, such as increased pelvic tilt, alteration of lumbar lordosis [13], decreased sacral slope, and spinopelvic mismatch as sagittal spinopelvic malalignment [14] like the increased Tönnis angle, Wiberg angle, or acetabular extrusion index.

Biological factors may also play a role in joint destruction in RPOH, but their exact contribution remains to be elucidated. Recently, there has been talk of inflammasome [15], a multiprotein complex responsible for the activation of inflammatory processes.

From the interaction of three factors: mechanical stress, cartilage degeneration, and bone response to the destructive process and its speed of progression, we could have two scenarios [16]. Mechanical stress, without activation of the inflammasome, associated with slow cartilage degeneration, accompanied by an adequate bone response, generates the appearance of hypertrophic osteoarthritis, with sclerosis and osteophytes; however, mechanical stress associated with activation of the inflammasome will generate rapid degeneration of the cartilage, with a slow reparative bone response, which will produce the appearance of destructive osteoarthritis, initially characterized by being initially atrophic in response, with minimal osteophytes.

The use of some drugs has also been related to the appearance of this clinical picture. Non-steroidal anti-inflammatory drugs have been cited and have specifically been related to the use of indomethacin. The use of corticosteroids has not been described as a factor favoring the disease, but various works have suggested the relationship between the RPOH and intra-articular corticosteroid injection. Boutin RD et al. found that 7% of patients receiving intra-articular infiltration with corticosteroid corticosteroids developed RPOH; although, these patients had special characteristics: older average age of patients with primary coxarthrosis, narrower joint space, and severe degenerative changes [17]. It is recommended to avoid intra-articular infiltration with corticosteroids in coxarthrosis with severe degenerative changes and always perform them in doses lower than 40 mg. Some authors, such as Okike, suggest a high risk of association in doses greater than 80 mg in one or multiple injections, but not in doses lower than 40 mg [18].

There has also been speculation about the waiting time to undergo surgery as a factor to be assessed in the development of this disease [19]; in our series, the periods from diagnosis to surgical repair were short. The national registry in England [20] suggests an increased incidence of RPOH, which may be related to longer waiting lists, given that the time from symptoms to destruction can be as short as 12 months [20,21,22].

Although everything seems to support the classic idea that we are faced with a process with inflammatory etiopathogenesis proposed by Conrozier [23] and with an autoimmune process as proposed by Tamai [24], all these biological factors may play a role in joint destruction in RPOH; however, their exact contribution is incompletely understood.

Pathologically, RPOH presents differential histological changes with respect to primary osteoarthritis and avascular necrosis, synovial infiltration of macrophages and osteoclasts, and synoviocytes showing elevated markers of inflammasome activation, proinflammatory cytokines, and metalloproteases, a possible explanation for the rapid joint involvement. The anatomopathological study presents, like in our series, characteristic areas with viable healthy tissue (viable zone), next to focal loci of osteonecrosis in subchondral bone (necrotic zone), and areas in bone repair (reparative zone), with the absence of a demarcation between healthy and necrotic tissue. While in avascular necrosis, the initial process would be in the heart of the femoral head with progressive involvement toward the periphery, in RPOH, the initial lesion would be peripheral, at the synovial level, with posterior centripetal extension in the femoral head. Massive synovial proliferation is likely to be the cause of the disease as a trigger for the massive activation of osteoblastic cells [25].

There have been several attempts to classify the disease based on classic works, the function of bone repair, and the speed of evolution of the deterioration. We speak of RPOH being rapid—18 months of chondrolysis, with an annual bone loss of 10–15 mm per year; RPOH being moderate—18–30 months of chondrolysis, with annual bone loss of around 5–10 mm, and RPOH being delayed—a progression of 3–5 mm annually for 3–5 years, subsequently followed by rapid or moderate deterioration. In our series, we can classify all patients as being within the rapid RPOH group.

Most series are clinically characterized by moderate involvement, a fact that contrasts with our series, with an average VAS pain rating of approximately 8, great functional limitation, and most patients needing level 3 analgesics to try to control the pain in most patients without success.

The diagnosis of RPOH is radiological in most cases. The lesion is evident and requires a differential diagnosis of other destructive lesions [26]. Without a doubt, the objective is to diagnose this condition early and to establish a therapeutic plan. Nelson FR et al. described radiographic measurements to aid in the early detection of evolving RPOH [27]. Zazgyva et al. proposed clinical-radiological diagnostic criteria and a radiological grading of severity, although these criteria have not been validated outside this study [28].

Sugano et al. have described findings for early detection in a study with nuclear magnetic resonance, joint space narrowing observed radiographically, and a diffuse abnormal pattern of low intensity on T1WI and a high intensity on T2WI induced by a small subchondral lesion, which might be an early sign of RPOH [29]. Watanabe et al. were the first to describe the entire process of hip destruction from the beginning to the terminal stage and the associated RPOH resonance alterations, suggesting that fractures due to subchondral insufficiency of the femoral head may be a sign prior to surgery [30] and the destruction of the femoral head. Although there is no data to support it, the presentation of subchondral fractures due to insufficiency could be related to the underlying osteoporotic process. The age of patients and the predominance of women are also coincident with this fact.

Intraoperative findings have been described in which the anterosuperior portion of the acetabular labrum had inverted toward the joint space, with fractures observed due to subchondral insufficiency of the femoral heads just below the inverted labrum. This may be involved in the rapid narrowing of the joint space and in subchondral insufficiency fracture in RPOH. Nuclear magnetic resonance in the early stages of ARP shows femoral and acetabular bone edema [12].

Among the markers studied to characterize this entity are the urinary increase in Helix-II and CTX-II markers resulting from the degradation of the cartilage [31], although not specific to the condition, and the increase in the blood metalloproteases MMP-3 [32] and MMP-9 that could help in the differential diagnosis of primary osteoarthritis. Most cases cannot be diagnosed early; however, some authors suggest that MMP-3 may predict subsequent femoral head destruction at the time before its initiation [33].

An increase in serum cross-linking c-terminal telopeptide levels has also been observed compared with patients with primary osteoarthritis. Following the opinions of some authors [34], its use could be useful to identify and monitor patients with RPOH.

Yasuda et al. [33] have spoken of two types of RPOH in initial stages depending on the presence or absence of bone destruction, type 1 with radiological criteria of rapid progression of the reduction of the joint space without cephalic destruction, and type 2 with the destruction of this, and have seen a different behavior in the evolution of the bone turnover markers analyzed (alkaline phosphatase and tartrate-resistant acid phosphatase-5b). They could be useful for early diagnosis and preventive treatment.

The differential diagnosis includes inflammatory pathologies, infections, avascular necrosis, and oncological pathologies.

Our work presents the limitation of short-medium term post-surgical follow-up and does not present a control group of patients operated on for primary coxarthrosis with a different patient pattern, with no difference in symptomatic patients between sexes, although with a higher radiographic prevalence in men; with a lower average age than that of patients with RPOH.

The progressive and destructive characteristics mean that medical treatment is not effective and only surgical treatment, including total hip replacement, is an option.

The surgical approach used in the different studies has varied from posterolateral, transtrochanteric, and transgluteal. They are surgeries that are not free of difficulty and have a higher complication rate than arthroplasty surgeries for primary coxarthrosis; Yuasa et al. described three dislocations in 12 patients operated on within the first 9 months in their series [35]. Baba et al. described two dislocations in 27 patients who underwent surgery. The alteration of the spinopelvic angles, described by some authors as part of the etiopathogenetic mechanism of RPOH, could be one of the causes of the highest rate of dislocation [36]. In our cases, we used the posterolateral approach in 14 surgeries and the anterolateral approach in 2. Although a higher risk of dislocation has been described in the posterior approach and in the RPOH, we have not had any dislocation in our series. One patient underwent surgery with a dual-mobility implant due to spinopelvic rigidity associated with the presence of severe lumbar spondyloarthrosis.

The surgical time is described in the literature as having an average duration of approximately 90 min, a time above that required for primary hip prosthetic surgery [22], which in our series was around 78 min on average.

The published series refers to greater blood loss [37], which is often attributed to the bone edema associated with these injuries, and which in many cases extends to the intertrochanteric region [38]. In this case series, these data have given us very different results; none of our patients required blood transfusion despite the complex surgical processes. We believe that this fact is determined by current blood-saving techniques, among which it is worth highlighting the use of tranexamic acid, compared with a series from a few years ago that described this fact.

The type of implants used for treating these patients varies and, in many cases, involves associated technical procedures. The series published before 2010 are those with cemented implants, stem, and acetabulum, with a loosening rate between 13% and 23% in the short and medium term. Postel and Kerboull presented a series of 42 patients with a 3-year follow-up and a rate of acetabular radiolucency imaging of approximately 34% and a 13% rate of symptomatic loosening [3]. Usui et al. with a series similar to ours, reported prosthetic loosening at 8 years of 26.6% [39]. Minamijima et al. found that out of 23 cases, 15 cases (57%) were found to have a clear zone at the acetabular component. Loosening in 6 hips (23%) and revision rate of the socket in 3 hips (12%) were found in an average follow-up of 8 years and 10 months [40].

Although larger series have been published with cemented implants, there are some series with hybrid and cementless arthroplasties, with optimal results and a 6-year survival rate of 100% [6].

In those patients who, in addition to cephalic femoral involvement, also have acetabular involvement and deformity, various series have been published with different solutions to acetabular defects; revision systems such as the oblong acetabular component and Kerboull type plate for acetabular roof reconstruction have been used [36]. Along these lines, Kawai et al. used Kerboull-type acetabular reinforcement devices and grafts in all their reported cases [41], while Peters et al. reported the use of revision acetabulum in all their cases with good results in a series of eight cases in the medium-term follow-up [42]. In a more recent series, results have been published with cementless implants with a loosening rate of 16.5% at 5 years. Some authors have even proposed more complex techniques in patients with massive acetabular destruction using structural allograft, osteosynthesis materials, and double mobility systems [43]. Yuasa et al. showed that cemented or cementless total hip prostheses in patients with RPOH achieved a good medium-term result, comparable to that of patients undergoing surgery with primary osteoarthritis [35].

In our series, we lack medium and long-term follow-up, with 13 months of average follow-up. The acetabular implants in the patients in our series were completely cementless, using in one case a revision cup and in another a double mobility system with optimal functional results, an improvement in the average Harris scale of 40 points 3 months after surgery and 50 points 1 year after surgery. From the radiological point of view, there are data of osseointegration of all implants, without data suggestive of loosening.

## 5. Conclusions

RPOH constitutes a differentiated idiopathic entity within hip osteoarthritis characterized by rapid radiological evolution. There was an incidence of 3.17% for RPOH in our institution. It was more common in women with an average age of around 77 years, unilaterally in most cases. There was significant clinical involvement with severe functional limitation and an MHS average of 30 points. The characteristics of bone quality require different options for prosthetic implants, with more complex surgeries and good functional recovery.

Greater patient follow-up is necessary to know the survival of the implants in these patients with an unknown etiology.

## Figures and Tables

**Table 1:** Characteristics of patients included in our series. CVRF: cardiovascular risk factors; preHHS: Harris Hip Score preoperative; HHS Year: Harris Hip Score one-year post-surgery.

PATIENT										
	Age	Sex	BMI	ASA	Side	CVRF	Pre-HHS	Implant	Transfusion	HHS Year
1	84	woman	36.1	III	Left	DM, HTA	30	Cementless	no	80
2	81	woman	22.2	III	Right	DM, HTA	30	Cementless	no	85
3	64	woman	31	II	Right	No	40	Cementless	no	95
4	79	woman	26.94	II	Left	DLP	35	Cementless	no	90
5	53	woman	41.4	II	Left	DM, HTA	40	Cementless	no	85
6	71	woman	29.9	III	Left	No	30	Cementless	no	85
7	87	woman	31	II	Right	DM, HTA, DLP	25	Cementless	no	75
8	88	men	28.7	II	Left	No	20	Cemented	no	80
9	80	woman	23.8	II	Right	DLP	30	Cementless	no	85
10	73	men	31.5	III	Left	DM	30	Cementless	no	100
11	85	woman	31.4	II	Right	HTA	25	Cemented	no	70
12	81	men	31	III	Left	DM, THE, DLP	30	Cementless	no	85
13		men	36.4	III	Right	HTA, Smoke	25	Cementless	no	90
14	73	men	37.4	III	Left	HTA, Smoke	35	Cementless	no	90
15	83	woman	30.4	II	Right	HTA	30	Cemented	no	80
16	80	woman	31	III	Right	No	30	Cementless	no	80

## Data Availability

Dataset available on request from the authors.
